# Diagnostic Accuracy Study of an Oscillometric Ankle-Brachial Index in Peripheral Arterial Disease: The Influence of Oscillometric Errors and Calcified Legs

**DOI:** 10.1371/journal.pone.0167408

**Published:** 2016-11-29

**Authors:** Ángel Herráiz-Adillo, Vicente Martínez-Vizcaíno, Iván Cavero-Redondo, Celia Álvarez-Bueno, Miriam Garrido-Miguel, Blanca Notario-Pacheco

**Affiliations:** 1 Department of Primary Care, Health Service of Castilla-La Mancha (SESCAM), Tragacete, Cuenca, Spain; 2 Universidad de Castilla-La Mancha. Health and Social Research Center. Cuenca, Spain; 3 Universidad Autónoma de Chile, Facultad de Ciencias de la Salud, Talca, Chile; The University of Tokyo, JAPAN

## Abstract

**Background:**

Peripheral arterial disease (PAD) is an indicator of widespread atherosclerosis. However, most individuals with PAD, in spite of being at high cardiovascular risk, are asymptomatic. This fact, together with the limitations of the Doppler ankle-brachial index (ABI), contributes to PAD underdiagnose. The aim of this study was to compare oscillometric ABI and Doppler ABI to diagnose peripheral arterial disease, and also to examine the influence of oscillometric errors and calcified legs on the PAD diagnoses.

**Methods and Findings:**

We measured the ankle-brachial indexes of 90 volunteers (n = 180 legs, age 70 ± 14 years, 43% diabetics) using both oscillometer OMRON-M3 and Doppler. For concordance analyses we used the Bland and Altman method, and also estimated the intraclass correlation coefficient. Receiver Operating Characteristic Curves were used to examine the diagnostic performance of both methods. The ABI means were 1.06 ± 0.14 and 1.04 ± 0.16 (p = 0.034) measured by oscillometer and Doppler ABIs respectively, with limits of agreement of ± 0.20 and intraclass correlation coefficient = 0.769. Oscillometer yielded 23 “error” measurements, and also overestimated the measurements in low ankle pressures. Using Doppler as gold standard, oscillometer performance for diagnosis of PAD showed an Area Under Curve = 0.944 (sensitivity: 66.7%, specificity: 96.8%). Moreover, when considered calcified legs and oscillometric “error” readings as arteriopathy equivalents, sensitivity rose to 78.2%, maintaining specificity in 96%. The best oscillometer cut-off point was 0.96 (sensitivity: 87%, specificity: 91%, positive likelihood ratio: 9.66 and negative likelihood ratio: 0.14).

**Conclusion:**

Despite its limitations, oscillometric ABI could be a useful tool for the diagnosis of PAD, particularly when considering calcified legs and oscillometric “errors” readings as peripheral arterial disease equivalents.

## Introduction

Peripheral arterial disease (PAD) is a clinical indicator of widespread atherosclerosis that affects nearly one in five people over 65 years old [1], and is considered a strong predictor of cardiovascular (CV) morbidity and all-cause mortality [2]. However, up to 80% of the cases remain undiagnosed [3], maybe because only one third of patients have symptoms [4], or because of a poorly implemented screening as a standard procedure in Primary Care Health System. The treatment of CV risk factors in these silent patients would improve their overall CV risk.

Doppler device remains as the non-invasive gold-standard to measure ankle-brachial index (ABI) and to identify subjects with PAD [5]. It has also proven to be a good predictor of CV events and overall mortality [2, 6].

Compared with angiography, a cut-off ≤ 0.9 in Doppler ABI has shown a high pooled diagnostic accuracy (0.80), sensitivity (0.75), specificity (0.86) and area under the Receiver Operating Characteristic Curve (ROC) (0.87) for PAD diagnosis [7].

Although Doppler ABI is a non-invasive, accessible, and inexpensive test, simultaneous measurements of blood pressure in all four extremities are impractical. Besides, it is also a time consuming test which requires technical skills, thus impeding the routine use of Doppler ABI technique in general practice. These limitations could be overcome using ABI measured by oscillometry, as it is a simpler and faster fully automatic test, which does not suffer from observer biases, and requires no special training [8, 9], making it a more suitable technique for general use and mass screening.

The diagnostic accuracy of oscillometric ABI versus Doppler ABI is controversial. Thus, while some authors propose replacing Doppler by oscillometry [10, 11], a recent meta-analysis has reported high average specificity (96%), but only moderate sensitivity (69%) [12] for oscillometry compared with Doppler. In addition, some studies have reported poor agreement between ABI values determined by Doppler and oscillometry [13–15], most of them however neglecting those subjects in which the oscillometric method reports an “error” message or Doppler reports values suggesting calcification.

The main aim of this study was to estimate the diagnostic accuracy of oscillometric ABI compared with Doppler ABI to diagnose PAD. A secondary aim was to analyse the influence in diagnostic accuracy of individuals with oscillometric errors and those with Doppler values suggesting calcification.

## Materials and methods

### Design and participants

This is an observational study designed for comparing oscillometric ABI (index test) with Doppler ABI (reference standard), following the Standards for Reporting of Diagnosis Accuracy Group (STARD) [16] according to a prospective design, (S1 Chart).

The study, conducted from January to September 2015, included 90 participants from two clinical settings. The first group consists of 66 subjects over 18 years old selected by consecutive sampling among those attending for any reason to the Primary Care Centre of Tragacete (Cuenca, Spain). The second group consists of 24 subjects over 18 with suspected PAD (positive Edinburgh Claudication Questionnaire [17]), selected by consecutive sampling among those attending the Vascular Surgery Unit from The Virgen de la Luz Hospital (Cuenca, Spain).

Exclusion criteria were: i) ulceration or edema in the leg, ii) arm circumference < 24 cm or > 32 cm, iii) morbid obesity, iv) atrial fibrillation, v) inability to tolerate supine position, and vi) refusal to sign the informed consent.

All subjects were informed of the aims and procedures of the study both orally and in writing and were asked to sign the informed consent. This study was approved by the Clinic Investigation Ethic Committee of Health Area of Cuenca, (Spain). REG: 2015jPI0815.

### Sample size

For sample size estimation, we hypothesized that a positive likelihood ratio (LR+) > 7 and a negative likelihood ratio (LR–) < 0.5 would be clinically useful. According to Simel´s equation [18], the values of sensitivity and specificity reported by Verberk et al. in a meta-analysis [12] (69% and 96%, respectively) and an expected prevalence of PAD of 35%, the minimum sample size would be 45 diseased and 129 non-diseased patients (total = 174).

### Variables

The following variables were collected from the electronic medical records: sociodemographic data, smoking status (current, former or never smoker), diagnosis of diabetes, hypertension or dyslipidemia and the most recent value of creatinine (mg/dl). We also measured by standard procedures height, weight, body mass index, diameter of the arm, ankle and calf, heart rate and waist-hip ratio.

#### Ankle-brachial index

In brief, all participants rested supine for 10 minutes, and measurements by Doppler and oscillometry were randomly determined in order to reduce the differences caused by initial stress. There were not either a time interval or a clinical intervention between index test and reference standard. In both techniques, the distal edge of the cuff was placed 2 cm above the malleoli and the elbow flexure. In the oscillometric technique, the measurement sensor faced the brachial artery and the posterior tibial artery. When an error message was reported twice, the sensor faced the dorsalis pedis artery.

ABI was calculated as: highest Doppler ankle pressure (dorsalis pedis or posterior tibial) / highest Doppler pressure between both arms for Doppler ABI; and mean of leg oscillometric systolic pressures / mean of the oscillometric systolic pressures in the arm with the highest pressure for oscillometric ABI.

Ultrasonic Pocket Doppler-Edan-Sonotrax-Basic (Edan-Instruments®, Shenzhen, China) was used in Doppler technique, with an 8 MHz probe and mercury sphygmomanometer Riester diplomat-presameter® (Riester, Jungingen, Germany) with an adult cuff (arm circumference 24–32 cm). Doppler ABI measurements were performed according to Aboyans et al. [5]: right brachial, right posterior tibial, right dorsalis pedis, left posterior tibial, left dorsalis pedis, and left and right brachial artery (a new measurement to cushion the effect of initial stress on the first one). Right arm pressure was calculated as the average of the two measurements when there was a difference ≤ 10 mmHg. We considered the second measurement when the difference between the two of them was > 10 mmHg.

Automatic oscillometric device OMRON-M3 (HEM-7200-E-Omron Healthcare, Kyoto, Japan) with a pressure cuff of 146 x 446 mm (arm circumference: 22–32 cm) was used in oscillometric technique. This device has been validated for measuring blood pressure in the arm with an estimated accuracy of ± 3 mmHg [19], but has not been specifically designed for ABI measurements. Two simultaneous and consecutive measurements on the four extremities were made, with one-minute interval. When blood pressure was not detected, two new measurements were made with one minute interval. A “measurement error” was considered when blood pressure was not detected in any of the four measurements.

At baseline, devices were calibrated by Electromedicine Service of Virgen de la Luz Hospital. A single nurse, trained on the ABI measurement technique according to ACC/AHA guidelines for the management of patients with PAD [5], performed all the measurements.

No adverse events were reported from performing both oscillometric and Doppler ABI.

### Statistical analyses

The adjustment of the variables to the normal distribution was tested by both statistical (Kolmogorov-Smirnov test) and graphic procedures (normal probability plot), (S1 Fig). Quantitative and qualitative variables were compared using Student's t-test and Pearson X^2^, respectively. A two-sided p-value ≤ 0.05 was considered significant.

The relationship between Doppler ABI and oscillometric ABI was estimated using Pearson correlation coefficient, and also beta coefficient in a linear regression model adjusting for age, diameter of the ankle, heart rate, gender and height [5]. The agreement between Doppler ABI and oscillometric ABI was assessed by intraclass correlation coefficient (ICC) and Bland and Altman plot [20], with a 95% confidence interval (CI). Moreover, the degree of diagnostic agreement was assessed using weighted kappa coefficient categorizing ABI as normal, low, and calcification.

The relationship between blood pressure differences of the two diagnostic methods versus Doppler blood pressure values in the ankle was examined in order to test if the differences varied systematically in the range of ABI, representing these differences by a box plot.

Reliability of both methods was estimated using Bland and Altman plot and also ICC.

The diagnostic accuracy of the oscillometric method for PAD was assessed calculating sensitivity, specificity, positive predictive value (PPV), negative predictive value (NPV), positive likelihood ratio (LR+), negative likelihood ratio (LR–) and diagnostic odds ratios (DOR). Each leg was analysed as an independent observation (n = 180). The area under ROC curve (AUC) was estimated with a nonparametric empirical approach, and the curves were compared using the DeLong´s test [21]. The best overall cut-off was estimated using Youden index.

Statistical analyses were performed using IBM® SPSS® 20, Epidat ® 3.1 and MedCalc® 15.2.2 software.

## Results

**Fig 1:**
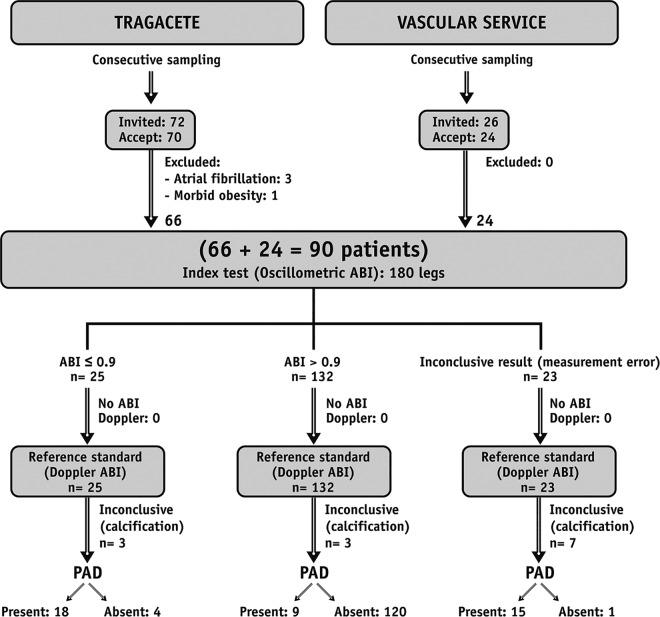
Flow chart of the study. Study design, number of participants and results of the measurements according to STARD [16] standards.

Fig 1 shows the flow chart of the study. Ninety participants (180 legs) were included. The characteristics of the study sample are presented in Table 1.

**Table 1:** Characteristics of participants by place of origin.

	Total	Primary Care Centre	Vascular Service	p
Subjects (n)	90	66	24	
Gender (% male)	62.2	56.1	79.2	0.005
Age (years)	70.4 ± 14.5	69.0 ± 15.9	74.1 ± 9.14	0.009
Weight (kg)	72.4 ± 15.0	71.5 ± 16.0	75.0 ± 10.9	0.111
Body mass index (kg/m^2^)	27.9 ± 4.68	27.6 ± 4.86	28.7 ± 4.03	0.200
Abdominal circumference (cm)	98.5 ± 13.7	96.9 ± 14.3	103.4 ± 10.3	0.007
Waist-hip ratio	0.97 ± 0.09	0.95 ± 0.09	1.00 ± 0.07	< 0.001
Heart rate	69.4 ± 12.6	68.3 ± 12.0	72.7 ± 13.8	0.042
Obesity, BMI ≥ 30 (%)	30	29	33	0.615
Hypertension (%)	69	64	83	0.012
Dyslipidemia (%)	62	55	83	< 0.001
Smoking status (%)	50	39	79	< 0.001
Diabetes (%)	43	30	79	< 0.001
Peripheral Arterial Disease (%)	23.3	12.1	54.2	< 0.001
Calcified legs (%)	7.2	4.5	14.6	0.021
**Maximum Doppler ABI**				
Total sample (n:180)	1.07 ± 0.35	1.11 ± 0.27	0.99 ± 0.50	0.118
Calcified legs excluded (n:167)	1.00 ± 0.21	1.06 ± 0.16	0.82 ± 0.22	< 0.001
Oscillometric errors and calcified legs excluded (n:151)	1.04 ± 0.16	1.09 ± 0.13	0.89 ± 0.18	< 0.001
**Oscillometric ABI**				
Total sample (n:180)	0.92 ± 0.37	1.02 ± 0.30	0.65 ± 0.44	< 0.001
Calcified legs excluded (n:167)	0.96 ± 0.34	1.04 ± 0.27	0.72 ± 0.41	< 0.001
Oscillometric errors and calcified legs excluded (n:151)	1.06 ± 0.14	1.10 ± 0.10	0.92 ± 0.15	< 0.001

Quantitative and qualitative variables are summarized as means ± standard deviations and percentages, respectively.

Sixty per cent of the legs (n = 108 legs, 47 subjects) assessed by Doppler had normal values (1.4 > ABI ≥ 1), 9% (n = 17 legs, 9 subjects) had borderline values (1 > ABI > 0.9), 22% (n = 40 legs, 24 subjects) had moderate PAD values (0.9 ≥ ABI ≥ 0.4) and 1% (n = 2 legs, 2 subjects) had severe PAD values (ABI < 0.4). Values suggesting arterial calcification (ABI ≥ 1.4) were found in 7.2% of the legs (n = 13 legs, 8 subjects).

It was impossible to measure blood pressure by oscillometry in 12.7% of the legs (n = 23 legs, 16 subjects). Of those, seven legs had arterial calcification, 15 had an ABI ≤ 0.9 and one had borderline ABI, with a mean of Doppler ABI in the last two groups of 0.62.

### Reliability

In a subsample of ten patients (20 legs) in which ABI was determined twice for each method, the average difference between the first and the second measurements for Doppler was 0.025 (95% CI: –0.012 to 0.062) and the ICC was 0.928 (95% CI: 0.830 to 0.971); and for oscillometer technique these estimates were 0.003 (95% CI: –0.034 to 0.04) and 0.956 (95% CI: 0.895 to 0.982), (S3 and S4 Figs).

### Comparison of pressures between methods in arms and ankles

Pearson correlation coefficient between Doppler and oscillometric pressures was 0.912 (p < 0.001) and 0.836 (p < 0.001) in arm and ankle, respectively (Table 2, S5 and S6 Figs). Compared with Doppler, the pressures in the arm were 1.05 mmHg (p = 0.114) lower when measured by oscillometry, and their limits of agreement of ± 17 mmHg, (S7 Fig); in the ankle, pressures were almost identical with limits of agreement of ± 27 mmHg, (S8 Fig).

**Table 2:** Correlation and level of agreement in the pressures of arm, ankle and ABI for Doppler and oscillometer.

	Pearson CC		Intraclass CC	Mean of differences (95% CI), mmHg (pressure) or Index
**Systolic arterial pressure (Arm)**				
**Total sample (n:180)**				
Osc right vs left	0.970*		0.985*	0.91 (0.18–1.64) ± 9.68*
Dop right vs left	0.942*		0.970*	4.06 (2.97–5.14) ± 14.37*
Osc vs Dop	0.912*		0.953*	–1.05 (–2.35–0.25) ± 17.32
**Systolic arterial pressure (Ankle)**				
**Osc vs Dop**				
Total sample (n:180)	0.141		0.247	–24.21 (–34.1–14.3) ± 131.7*
Calcified legs excluded (n:167)	0.793*		0.840*	–8.43 (–12.98- –3.88) ± 58*
Oscillometric errors and calcified legs excluded (n:151)	0.836*		0.902*	–0.15 (–2.39–2.09) ± 58
**Ankle Brachial Index**				
**Osc vs Dop** **ǂ**				
Total sample (n:180)	0.108	0.267 ƚ	0.108	–0.01 (–0.04–0.01) ± 0.38
Calcified legs excluded (n:167)	0.779*	0.754 ƚ	0.695*	–0.43 (–0.77- –0.01) ± 0.43*
Oscillometric errors and calcified legs excluded (n:151)	0.780*	0.747 ƚ	0.769*	0.02 (0.00–0.03) ± 0.20

CC: correlation coefficient; CI: Confidence Interval; Dop: Doppler; Osc: Oscillometer

*: P value < 0.001

ƚ: Partial correlation adjusting for age, gender, height and heart rate

ǂ: Doppler calculated as the highest pressure, posterior tibial or dorsalis pedis.

### Comparison of ABI measurements between methods

When calcified legs and oscillometric measurement errors were excluded (n = 151 measurements), Pearson correlation coefficient between Doppler and oscillometric ABI was 0.780 (p < 0.001), (S2 Fig), and it did not substantially change after controlling for age, ankle diameter, heart rate, gender and height (r = 0.747, p < 0.001). ICC between both measurement methods was 0.769 (p < 0.001).

**Fig 2:**
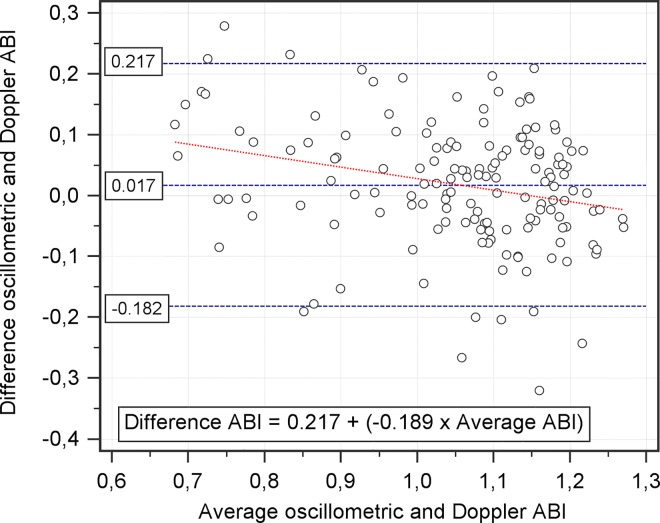
Bland and Altman plot of the oscillometric and Doppler ABI. The differences between both methods, oscillometric and Doppler, are plotted as a function of the average of the two methods (n = 151). The blue dashed lines show the mean difference with 95% CI. Red dashed line shows the linear regression.

**Fig 3:**
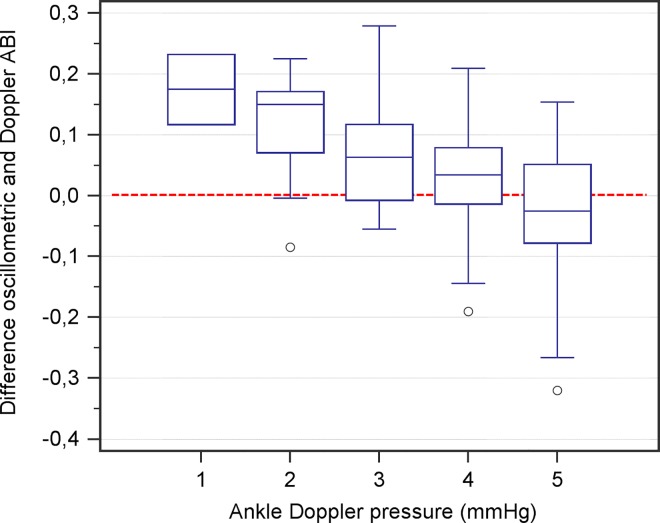
Box plots of the differences between methods in ABI according to ankle pressure. Differences between oscillometric and Doppler ABI values (Y axis) are plotted as a function of the ankle pressure in mmHg measured by Doppler (X axis). Measurements are divided in 5 groups according to ankle pressure (mmHg) in intervals of 20 mmHg: 1 (70–89 mmHg), 2 (90–109 mmHg), 3 (110–129 mmHg), 4 (130–149 mmHg) and 5 (> 150 mmHg).

Means of measurements of oscillometer and Doppler ABI were: 1.06 ± 0.14 and 1.04 ± 0.16 (p = 0.034), respectively. Bland and Altman plot showed a mean difference between methods of 0.017 with limits of agreement of ± 0.20 (p = 0.034), (Fig 2). A regression analysis showed that the differences between Doppler and oscillometry measurements varied depending on the range of ABI, with r = –0.261 (p = 0.001), (Figs 2 and 3). This measurement bias was far more pronounced in diabetic population (r = –0.448, p < 0.001, in diabetics; r = –0.017, p = 0.874, in non-diabetics).

### Diagnostic accuracy of oscillometric method

For PAD diagnosis, considering a cut-off of 0.9 in Doppler ABI as reference and when legs with either oscillometric measurement errors or calcification were excluded (n = 151 legs), oscillometric ABI showed sensitivity of 66.7%, specificity of 96.8%, PPV of 81.8% and NPV of 93.0%, with AUC = 0.944 (95% CI: 0.905 to 0.983) (S1 Table). Kappa coefficient showed a value of 0.684.

When oscillometric measurement errors were considered as PAD equivalents (n = 167 legs), sensitivity increased to 78.6%, maintaining specificity in 96.0%, with AUC = 0.958 (95% CI: 0.928 to 0.987) and kappa coefficient of 0.77, (S2 Table).

**Fig 4:**
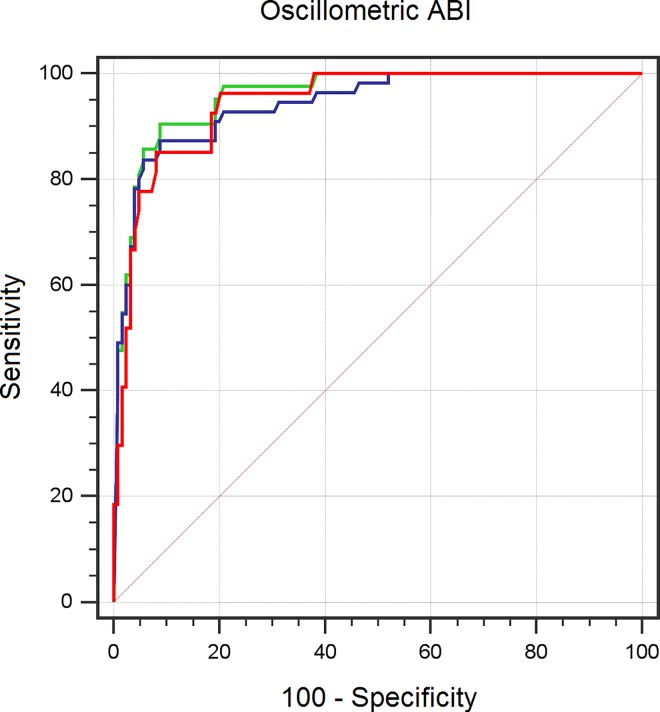
ROC curve for determining PAD by oscillometric ABI compared with Doppler. Blue line: total sample, n = 180. Green line: calcified legs are excluded; oscillometric measurement errors are included, n = 167. Red line: oscillometric measurement errors and calcified legs are excluded, n = 151. The areas under the curve were 0.914, 0.958 and 0.944 respectively, showing the DeLong´s test no statistically significant differences between them.

Additionally, when considering calcified legs as PAD equivalents (n = 180 legs) [22], sensitivity and specificity were maintained (78.2% and 96%, respectively), with kappa coefficient of 0.645 and AUC = 0.914 (95% CI: 0.872 to 0.955), (S3 Table). No statistically significant differences between the three AUCs were found. Dichotomous kappa (healthy and diseased: ABI < 0.9 and calcified) was 0.769, (Table 3 and Fig 4).

**Fig 5:**
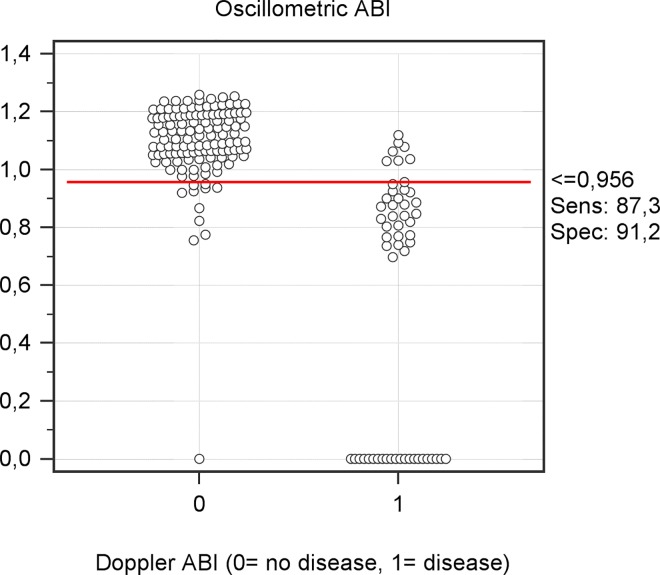
Interactive dot diagram of oscillometer and Doppler ABI. It depicts how the best cut-off (0.956) classifies oscillometric measurements regarding Doppler ABI as well as measurement errors. There were 23 oscillometric measurement errors, of those, only one had a normal ABI.

The best overall cut-off for oscillometric ABI, estimated by Youden index was = 0.96 (sensitivity 85%, specificity 92%), (Fig 5 and S9 Fig).

**Table 3:** Diagnostic performance of oscillometric ABI in the detection of PAD compared with Doppler ABI.

Cut-off Oscillometric ABI	Sensitivity*	Specificity*	PPV*	NPV*	LR+*	LR–*	DOR	Accuracy
0.9								
Oscillometric errors and calcified legs excluded (n:151)	66.7	96.8	81.8	93	20.67	0.34	60.79	91.39
	(47.0–86.3)	(93.3–100)	(63.4–100)	(88.2–97.8)	(7.6–56.2)	(0.2–0.59)		
Calcified legs excluded (n:167)	78.6	96	86.8	93	19.64	0.22	89.27	91.62
	(65.0–92.2)	(92.2–99.8)	(74.8–98.9)	(88.2–97.8)	(8.2–47.0)	(0.12–0.40)		
Total sample (n:180)	78.2	96	89.6	90.9	19.55	0.23	85	90.56
	(66.4–90)	(92.2–99.8)	(79.9–99.3)	(85.6–96.2)	(8.2–46.7)	(0.14–0.38)		
1.0								
Oscillometric errors and calcified legs excluded (n:151)	85.2	88.7	62.2	96.5	7.54	0.17	44.35	88.08
	(69.9–100)	(82.7–94.7)	(45.2–79.1)	(92.7–100)	(4.5–12.7)	(0.07–0.41)		
Calcified legs excluded (n:167)	90.5	88	71.7	96.5	7.54	0.11	68.54	88.62
	(80.4–100)	(81.9–94.1)	(58.6–84.8)	(92.7–100)	(4.64–12.2)	(0.04–0.28)		
Total sample (n:180)	90.5	81.9	60.3	96.6	4.99	0.12	41.58	83.89
	(80.4–100)	(75.1–88.7)	(47.4–73.2)	(92.9–100)	(3.46–7.22)	(0.05–0.3)		
1.1								
Oscillometric errors and calcified legs excluded (n:151)	100	52.4	31.4	100	2.1	-	-	60.93
	(98.1–100)	(43.2–61.6)	(21–41.8)	(99.2–100)	(1.75–2.53)			
Calcified legs excluded (n:167)	100	52	41.2	100	2.08	-	-	64.07
	(98.8–100)	(42.8–61.2)	(31.1–51.2)	(99.2–100)	(1.74–2.5)			
Total sample (n:180)	100	47.8	36.8	100	1.92	-	-	60.00
	(98.8–100)	(39.1–56.5)	(27.5–46.1)	(99.2–100)	(1.63–2.25)			

ABI: ankle brachial index; DOR: Diagnostic Odds Ratio; LR–: Negative Likelihood Ratio; LR+: Positive Likelihood Ratio; NPV: Negative Predictive Value; PPV: Positive Predictive Value

*: 95% Confidence Intervals are included.

## Discussion

The main aim of this study was to examine the accuracy of ABI measured by an automatic oscillometer to diagnose PAD. Oscillometric ABI showed an acceptable sensitivity (78.2%), excellent specificity (96%) and good diagnostic performance (DOR = 85) compared with Doppler ABI (non-invasive gold-standard) to diagnose PAD. Moreover, there was good diagnostic agreement between both methods. Thus, ABI measured by the oscillometric method, because of its simplicity and applicability, might be a useful tool for screening and diagnosis of PAD in Primary Care Settings.

A secondary aim was to analyze the influence of oscillometric measurement errors and calcified legs in diagnostic performance. Oscillometric measurement errors and calcified legs have already been reported but, to our knowledge, the present study is the first attempt to formally analyze its influence in diagnostic performance. It has been suggested that calcified legs [22] or an error in the oscillometric measurement [9, 23] should be interpreted as PAD equivalents. Most of calcified legs have PAD, and all of them could be considered at high CV risk. Thus, in our study, all but one of the 23 subjects in which it was not possible to measure ankle pressure (reported as oscillometric error) showed either low ABI or calcification. To detect PAD and considering oscillometric errors and calcified legs as PAD equivalents, specificity is maintained while sensitivity rises from 66.7% to 78.2%, with a DOR value of 85. Besides, the concordance between Doppler and oscillometric method remains excellent (dichotomous kappa = 0.77). When excluding calcified legs in the analysis (n = 167), the method did show the best diagnostic performance (DOR = 89.27).

In our study, oscillometric ABI did not detect calcification (as ABI > 1.4) in any case, but reported an error message with a sensitivity of 77% and 100% specificity. Both oscillometric and Doppler ABI have important limitations in calcified patients. Thus, it would be interesting to compare oscillometry with toe-brachial index [24] or pulse volume recording (PVR)—two techniques that have proved to be reliable in non-compressible vessels—to know the role of oscillometry in such patients. However, both techniques might be unpractical outside vascular laboratories. In our study, probably due to a high prevalence of diabetes (43%), a high percentage of calcified legs was found, so the estimation of the influence of calcification in the analysis may be overestimated.

In addition to low ABI or calcification, there are other reasons that can explain the aforementioned oscillometric errors, such us arrhythmia, movement during measurement or inaccurate wrapping of cuff. In all these situations, the device displays different specific icons of error. Thus, in the presence of an oscillometric error along with any specific icons of error, it would be appropriate to confirm the results with the Doppler technique to avoid a false positive PAD diagnosis. This precaution also extends to those patients in whom low cardiac ejection function is suspected, as it is also a potential cause of measurement error.

Usually, a diagnostic test is considered excellent when it exhibits a LR+ > 10 and LR–< 0.1 [25]. In the present study, a high specificity and LR+ > 10 (19.55) largely indicate the ability of the oscillometry to confirm the presence of PAD. By contrast, an only moderate sensitivity and LR–> 0.1 (0.23), indicate that the test has only a moderate ability to rule out the disease (when the test result is negative).

Consistent with other studies, the best overall cut-off for oscillometric ABI in the diagnosis of PAD was above 0.9 [9, 14, 26]. Using this cut-off and when considering the total population (including oscillometric errors and calcified legs), the diagnosis performance of the test might be considered acceptable (cut-off = 0.96, sensitivity 87%, specificity 91%). In a population aged 65 and over, in which the reported prevalence is 18% [1], the PPV and NPV would be 68% and 97%, respectively. Thus, in a real context of Primary Care practices, the clinical value of the test to rule out the disease would be even greater than their ability to confirm it, demonstrating the usefulness of oscillometric ABI in screening for PAD. In a scenario with a lower prevalence of PAD (i.e. people younger than 65 years), these capacity to rule out PAD would be even more remarkable, because NPV will increase with decreasing prevalence.

In general, agreement between oscillometric ABI and Doppler ABI was good (ICC = 0.769) and the test-retest reliability of oscillometric ABI was similar to Doppler ABI. In addition, the mean difference between oscillometric ABI and Doppler ABI (0.017 with limits of agreement of ± 0.20), is similar to that reported in other validation studies of similar devices [11, 26–28].

Also consistent with other studies [28, 29], a major oscillometer drawback is that pressure difference between both methods in ankle varies significantly according to the pressure range, showing an inverse relationship in such a way that at pressures below 110 mmHg the oscillometer overestimated the pressure up to 25 mmHg, with a potential loss of sensitivity. This measurement bias preferably occurs in the diabetic population. However, this under or overestimation in ABI by the oscillometer technique especially happens at extreme values of ABI, thus does not affect the agreement on the area of discrimination (0.9). Therefore, our data support that oscillometry seems to be a valid technique to diagnosis PAD, but not its severity degree.

Another oscillometer drawback, also reported in other studies [8, 23, 26], is its lack of ability to measure low pressures in the ankle in comparison to Doppler. This was responsible for obtaining 23 measurement errors with oscillometric technique, which despite giving useful information either low ABI or calcification in such patients, would invalidate the use of this device to monitor the evolution of those with severe PAD.

Despite these limitations, oscillometric ankle-brachial index might be a useful tool to screen and diagnose PAD. Our results suggest a mismatch between traditional results and those considering oscillometric errors and calcified legs. At least from a CV risk prevention perspective, this could be important, because traditional oscillometric sensitivity and specificity may have been undervalued to detect high CV risk patients.

### Study limitations

This study has some limitations that must be taken into account. Firstly, in this study we used Doppler ABI as reference standard. Although it is considered the non-invasive gold-standard, it has some limitations, especially in non-expert hands [10]. Ideally, oscillometric ABI should be compared with digital subtraction angiography, but such an invasive technique in a population without very high CV risk profile or revascularization expectations could be ethically unjustifiable. Secondly, our design with a unique examiner and a randomized order of measurement let us mitigate the initial effect of stress in both techniques equally, as well as eliminate inter-observer variability. However, due to the presence of a unique examiner, it was not possible to blind the measurements between oscillometric and Doppler technique. As the oscillometric technique is a fully automatic technique, a bias is only permissible—diagnostic review bias—when the oscillometric technique was performed prior to Doppler. Despite that, the analysis of the mean values of Doppler ABI, differences between techniques and likelihood ratios showed no statistically significant differences between both groups according to the order of measurement. Thirdly, although less variability has been reported with multiple measurements, in this study only one Doppler measurement was performed in each artery; in the right arm, however, two measurements were made to mitigate the effect of initial stress. Fourthly, we used an oscillometric device which has not been designed for neither ABI measurement nor blood pressure measurement in the lower limb; however, some meta-analysis [12, 30] have provided evidence supporting that simultaneous measurements with regular oscillometers might achieve enough accuracy in the measurement of arm and ankle blood pressures.

## Supporting Information

S1 Chart STARD checklist of the study. (DOCX)

S1 Fig Normal probability plot. Distribution of the differences between oscillometric and Doppler ankle-brachial index readings.
(TIF)

S2 Fig Scatter plot of oscillometric and Doppler ABI. The solid line shows the best regression line with 95% confidence interval, n = 151.
(TIF)

S3 Fig Bland and Altman plot testing for reliability in Doppler. Ten subjects (20 legs) were examined twice with Doppler to calculate ABI. The differences between the first and the second measurements are plotted as a function of the average of the two measurements (n = 20). The solid line shows the mean difference with 95% confidence intervals.
(TIF)

S4 Fig Bland and Altman plot testing for reliability in oscillometer. Ten subjects (20 legs) were examined twice with oscillometer to calculate ABI. The differences between first and second measurements are plotted as a function of the average of the two measurements (n = 20). The solid line shows the mean difference with 95% confidence intervals.
(TIF)

S5 Fig Scatter plot of oscillometer vs Doppler pressures in the arm. The solid line shows the best regression line with 95% confidence interval. The equation shows the oscillometer pressure as a function of Doppler pressure.
(TIF)

S6 Fig Scatter plot of oscillometer vs Doppler pressures in the ankle. The solid line shows the best regression line with 95% confidence interval. The equation shows the oscillometer pressure as a function of Doppler pressure.
(TIF)

S7 Fig Bland and Altman plot of oscillometer versus Doppler pressures in the arm. The differences between methods are plotted as a function of the average of the two methods. The solid line shows the mean difference with 95% confidence intervals.
(TIF)

S8 Fig Bland and Altman plot of oscillometer versus Doppler pressures in the ankle. The differences between methods are plotted as a function of the average of the two methods. The solid line shows the mean difference with 95% confidence intervals.
(TIF)

S9 Fig Best cut-off determination for maximizing both the sensitivity and the specificity. The best oscillometric cut-off was 0.956, with 87.3% of sensitivity and 91.2% or specificity.
(TIF)

S1 Table 2x2 Table of reference standard (Doppler) and index test (oscillometer) in ABI determination, excluding oscillometric errors and calcified legs. Each leg is analyzed separately, thus making each leg an independent observation.
(PDF)

S2 Table 2x2 Table of reference standard (Doppler) and index test (oscillometer) in ABI determination, excluding calcified legs and considering oscillometric errors as PAD equivalents. Each leg is analyzed separately, thus making each leg an independent observation.
(PDF)

S3 Table 2x2 Table of reference standard (Doppler) and index test (oscillometer) in ABI determination, including calcified legs and oscillometric errors as PAD equivalents. Each leg is analyzed separately, thus making each leg an independent observation.
(PDF)
