# Supplementary figures and images for: Diagnostic Accuracy Study of an Oscillometric Ankle-Brachial Index in Peripheral Arterial Disease: The Influence of Oscillometric Errors and Calcified Legs
Source: PLoS One. 2016 Nov 29;11(11):e0167408. doi: 10.1371/journal.pone.0167408 (PMC5127576; doi:10.1371/journal.pone.0167408)

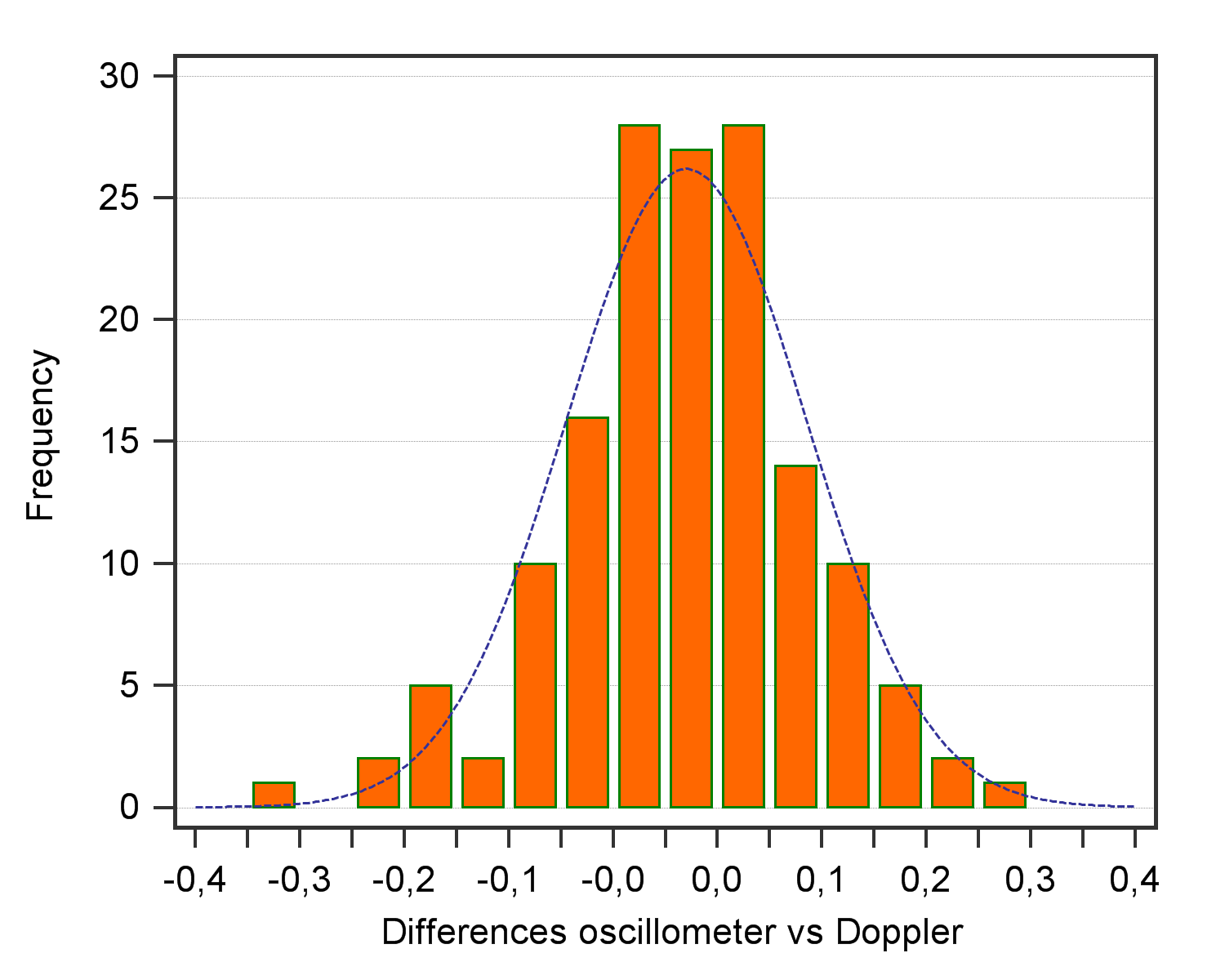

Supplement: S1 Fig — Distribution of the differences between oscillometric and Doppler ankle-brachial index readings. (TIF) [file pone.0167408.s002.tif]

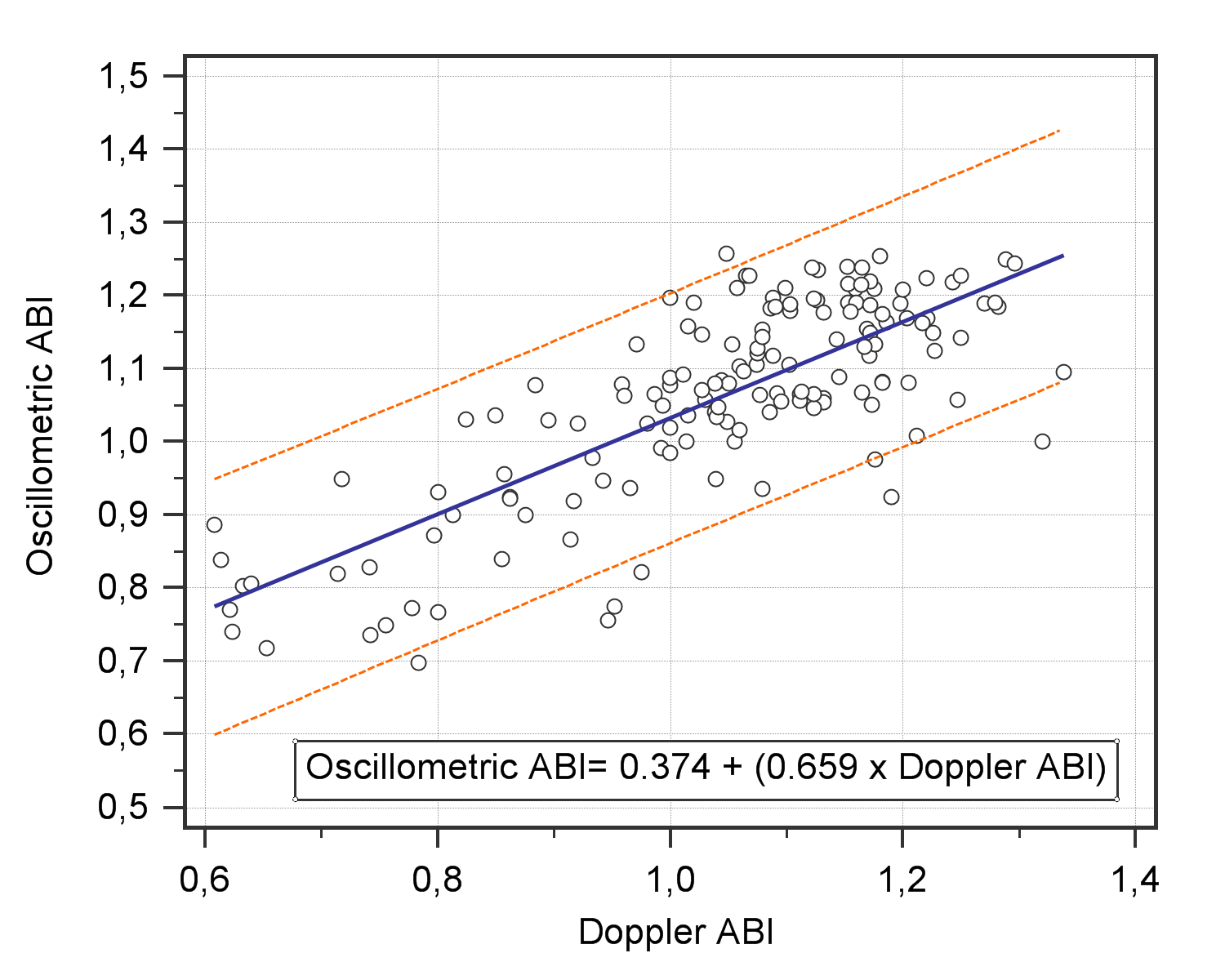

Supplement: S2 Fig — The solid line shows the best regression line with 95% confidence interval, n = 151. (TIF) [file pone.0167408.s003.tif]

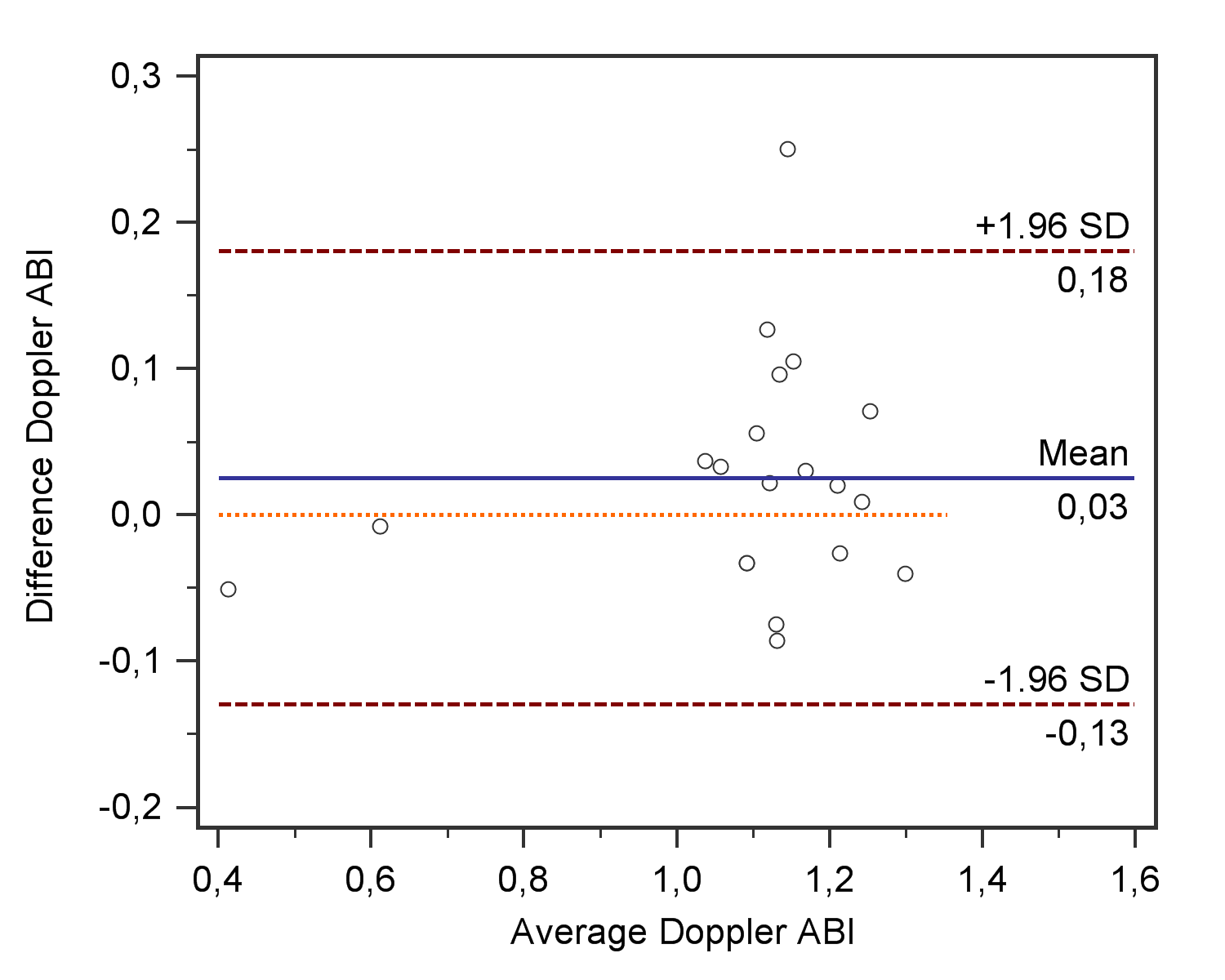

Supplement: S3 Fig — Ten subjects (20 legs) were examined twice with Doppler to calculate ABI. The differences between the first and the second measurements are plotted as a function of the average of the two measurements (n = 20). The solid line shows the mean difference with 95% confidence intervals. (TIF) [file pone.0167408.s004.tif]

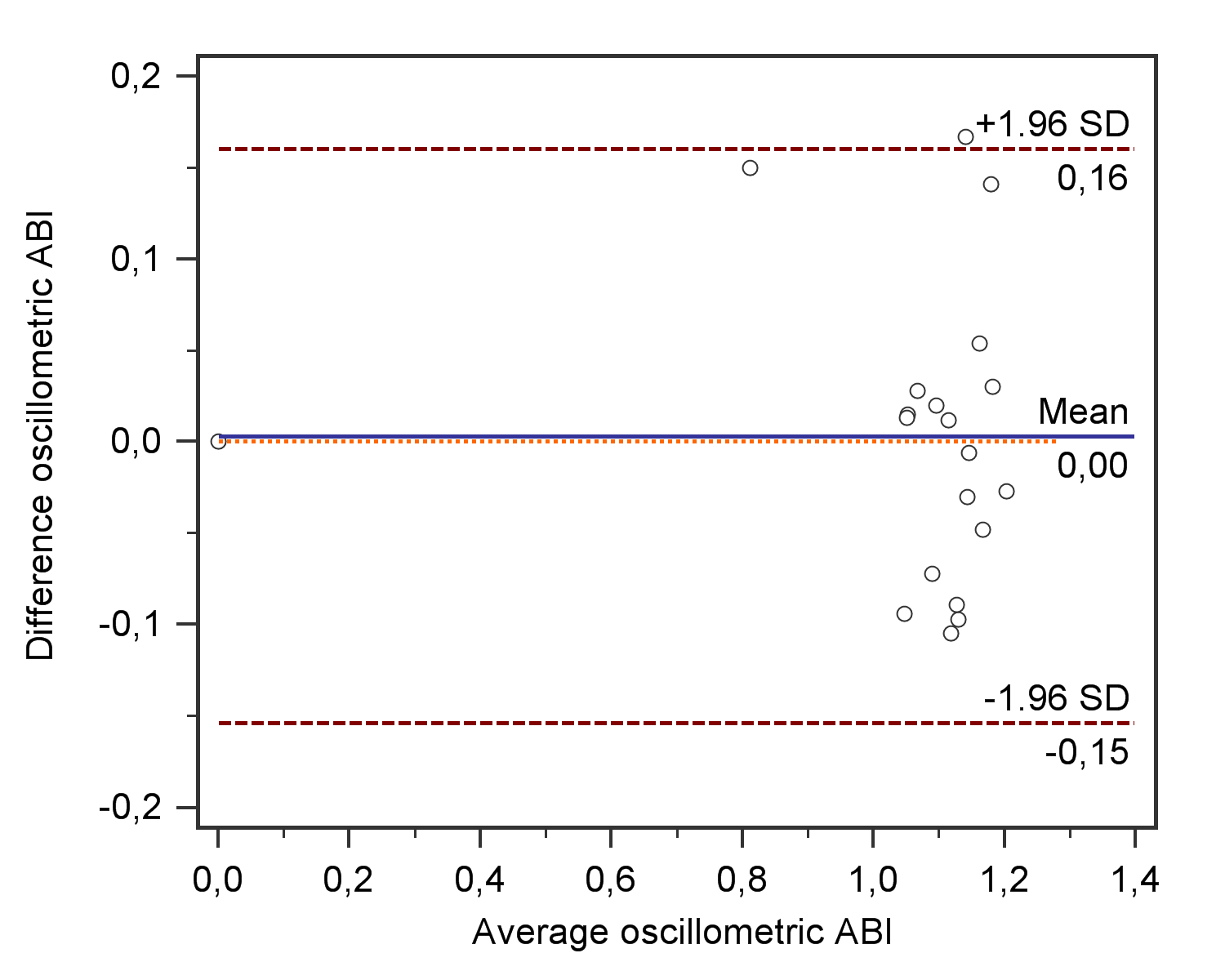

Supplement: S4 Fig — Ten subjects (20 legs) were examined twice with oscillometer to calculate ABI. The differences between first and second measurements are plotted as a function of the average of the two measurements (n = 20). The solid line shows the mean difference with 95% confidence intervals. (TIF) [file pone.0167408.s005.tif]

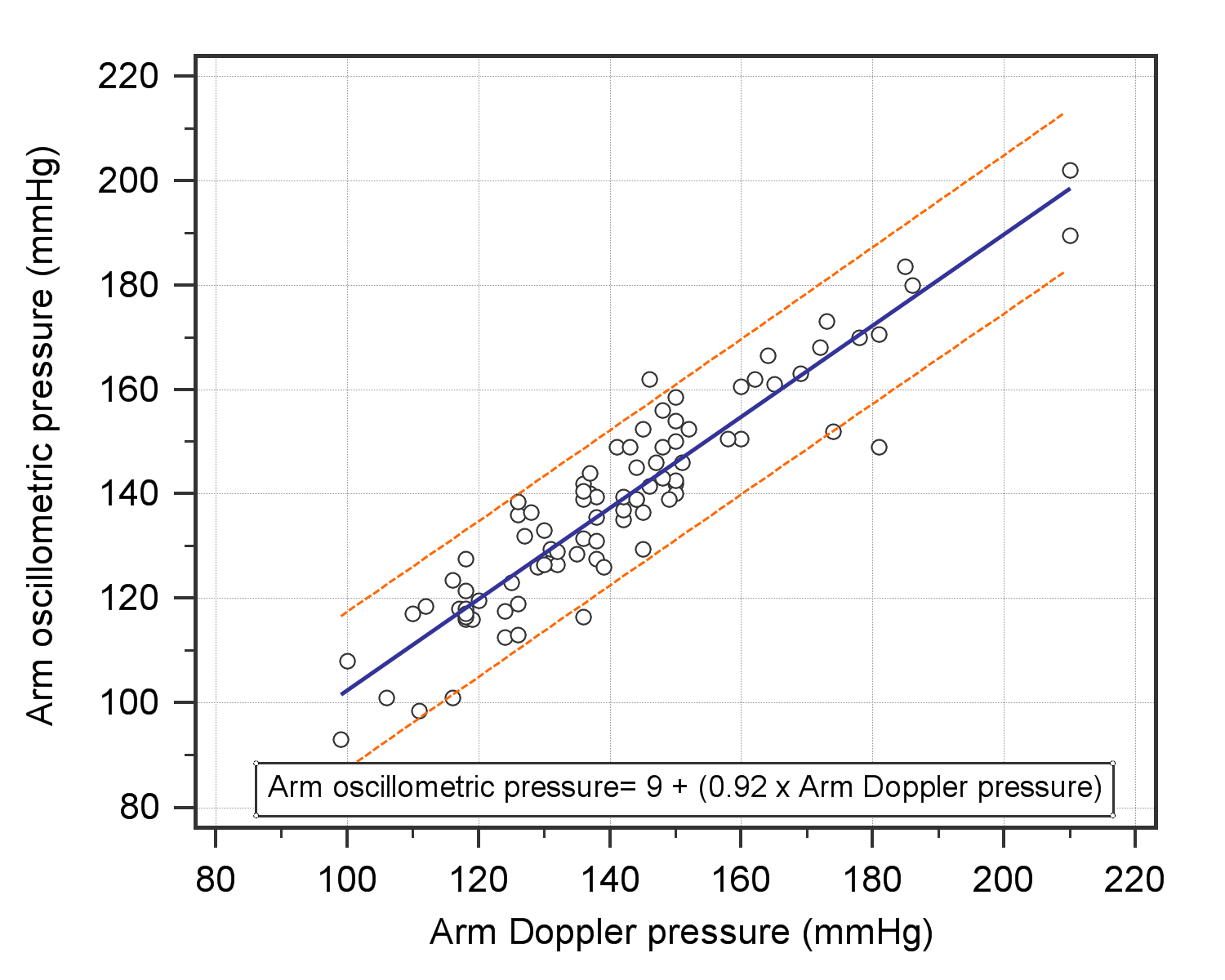

Supplement: S5 Fig — The solid line shows the best regression line with 95% confidence interval. The equation shows the oscillometer pressure as a function of Doppler pressure. (TIF) [file pone.0167408.s006.tif]

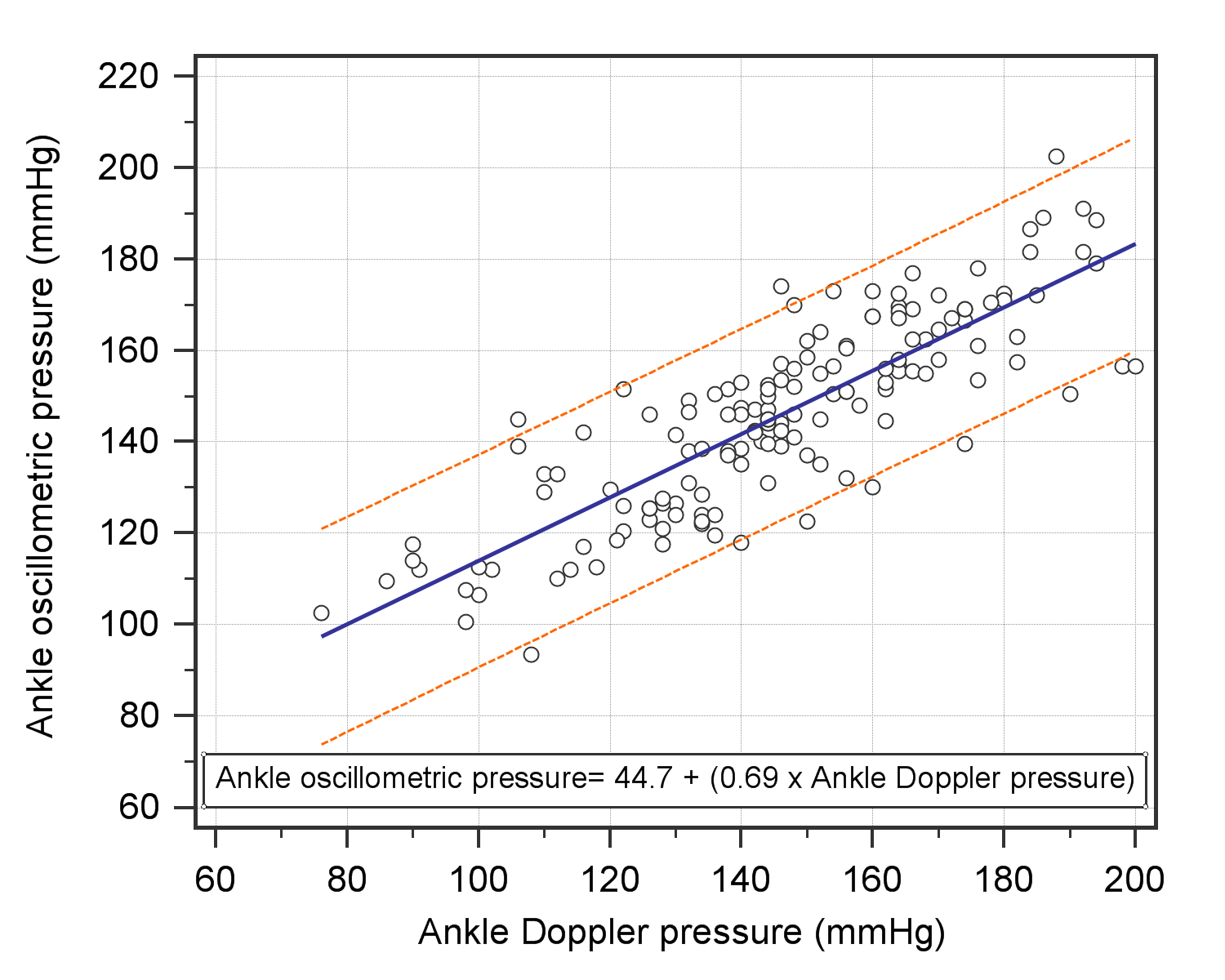

Supplement: S6 Fig — The solid line shows the best regression line with 95% confidence interval. The equation shows the oscillometer pressure as a function of Doppler pressure. (TIF) [file pone.0167408.s007.tif]

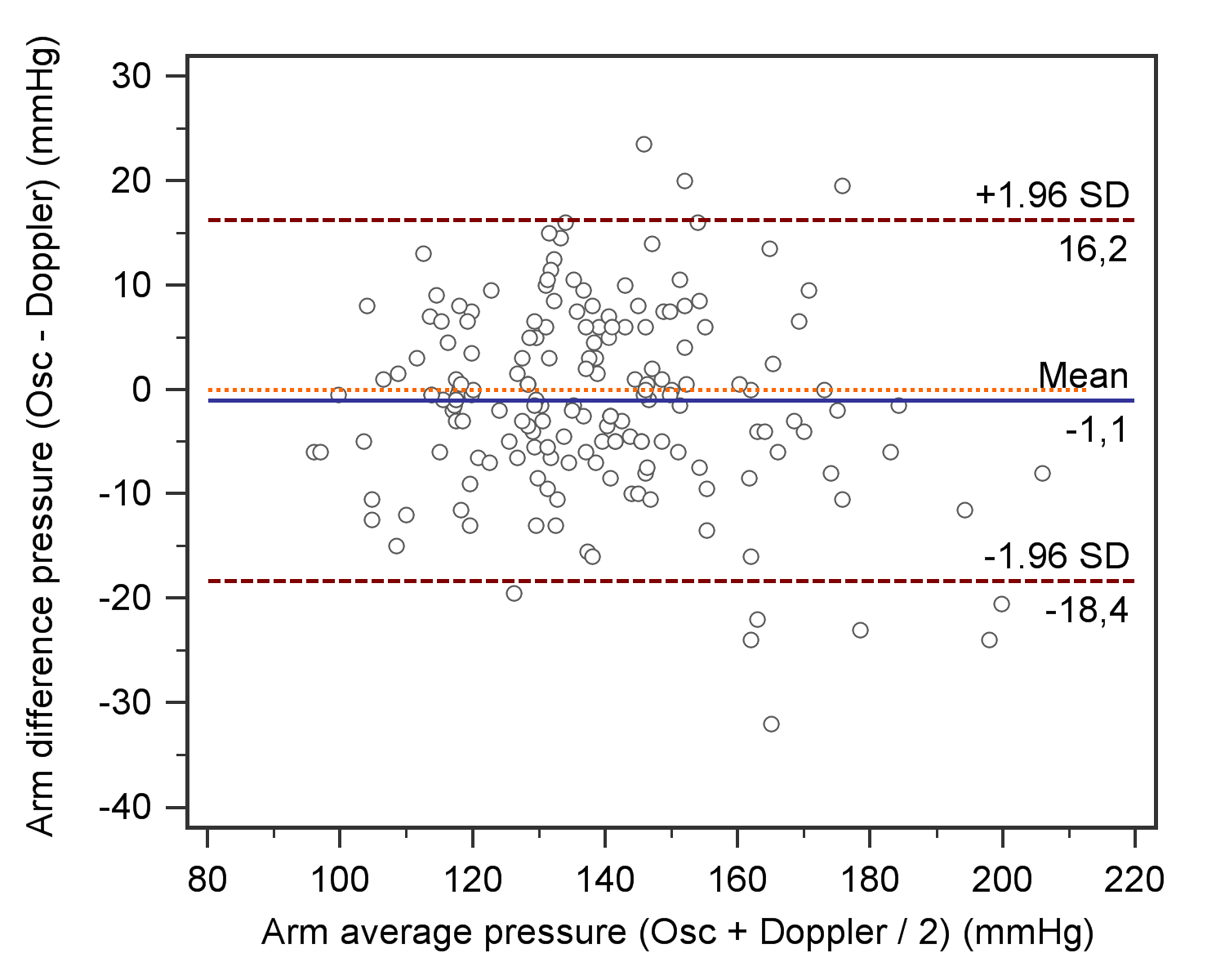

Supplement: S7 Fig — The differences between methods are plotted as a function of the average of the two methods. The solid line shows the mean difference with 95% confidence intervals. (TIF) [file pone.0167408.s008.tif]

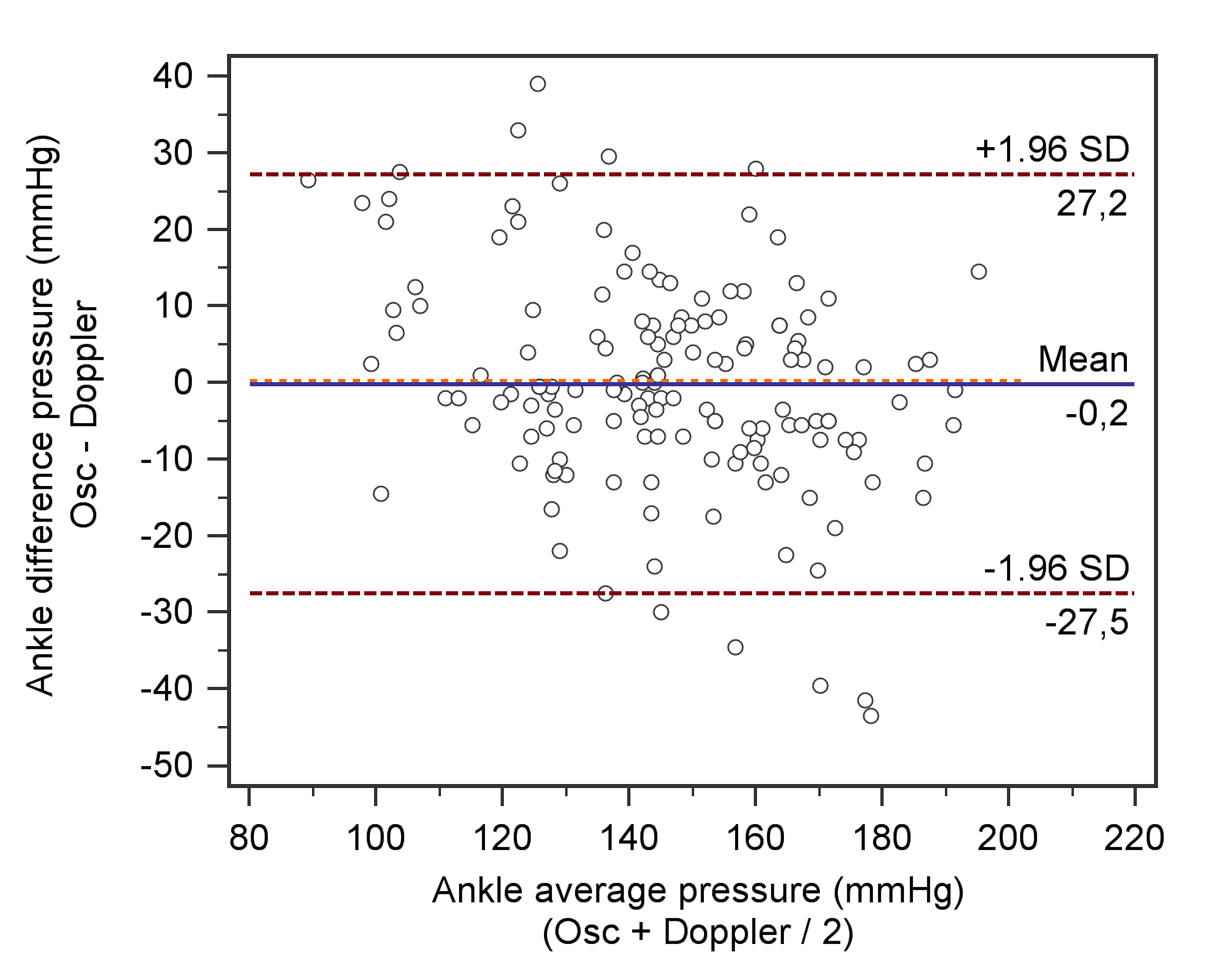

Supplement: S8 Fig — The differences between methods are plotted as a function of the average of the two methods. The solid line shows the mean difference with 95% confidence intervals. (TIF) [file pone.0167408.s009.tif]

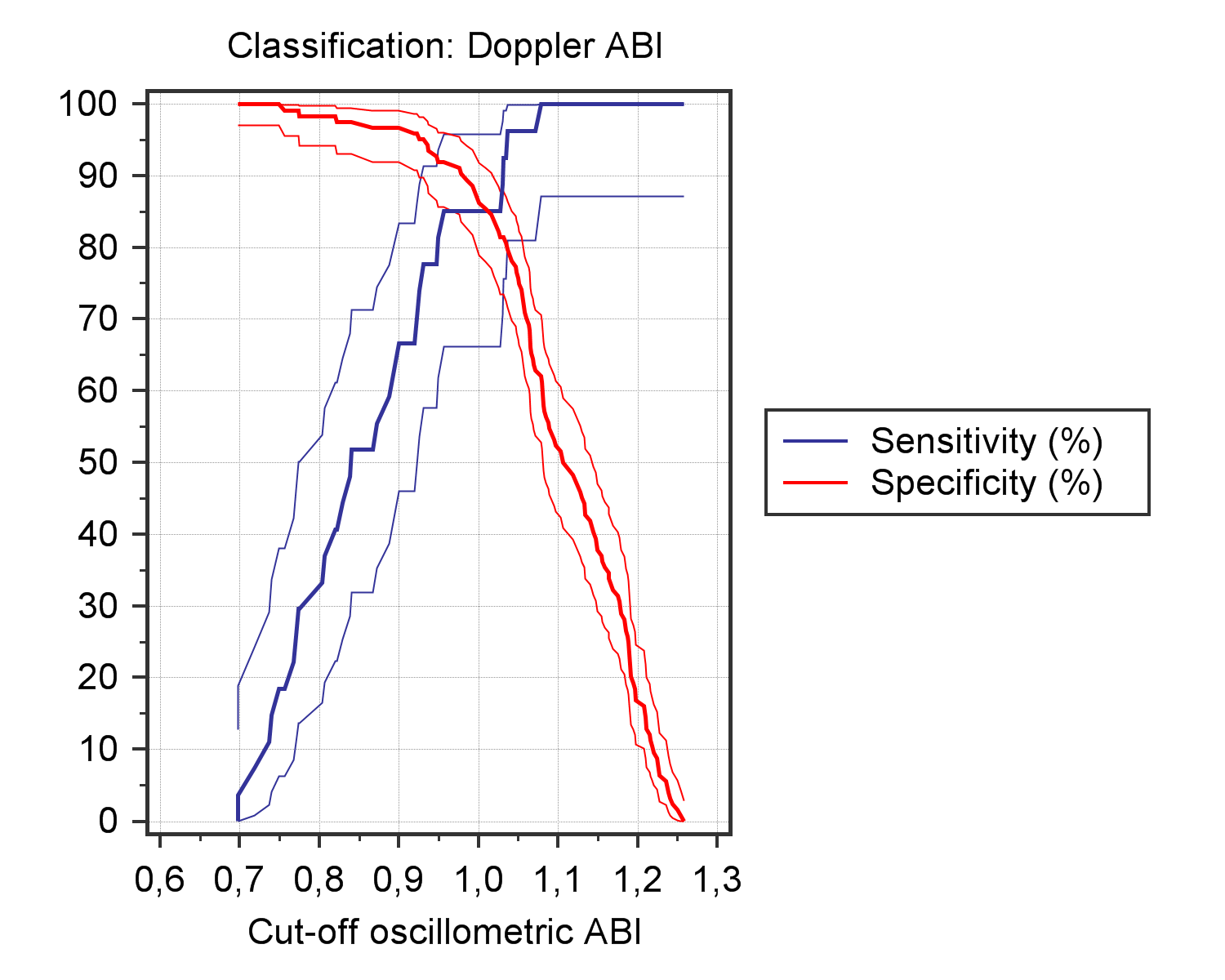

Supplement: S9 Fig — The best oscillometric cut-off was 0.956, with 87.3% of sensitivity and 91.2% or specificity. (TIF) [file pone.0167408.s010.tif]
