# Supplementary material for: Diagnostic Accuracy Study of an Oscillometric Ankle-Brachial Index in Peripheral Arterial Disease: The Influence of Oscillometric Errors and Calcified Legs
Source: PLoS One. 2016 Nov 29;11(11):e0167408. doi: 10.1371/journal.pone.0167408 (PMC5127576; doi:10.1371/journal.pone.0167408)
Supplement: S1 Table — Each leg is analyzed separately, thus making each leg an independent observation. (PDF) [file pone.0167408.s011.pdf]

	**PAD positive** (Doppler ABI < 0.9)	**PAD negative** (Doppler ABI ≥ 0.9)	**Total**
**Test positive** (Oscillometric ABI < 0.9)	18	4	22
**Test negative** (Oscillometric ABI ≥ 0.9)	9	120	129
**Total**	27	124	151
